# The planarian *TRPA1* homolog mediates extraocular behavioral responses to near-ultraviolet light

**DOI:** 10.1242/jeb.152298

**Published:** 2017-07-15

**Authors:** Taylor R. Birkholz, Wendy S. Beane

**Affiliations:** Department of Biological Sciences, Western Michigan University, 1903 W. Michigan Avenue, Kalamazoo, MI 49008, USA

**Keywords:** Planaria, Extraocular photoreception, Dermal phototransduction, UV-light detection, TRPA1, Neuroethology

## Abstract

Although light is most commonly thought of as a visual cue, many animals possess mechanisms to detect light outside of the eye for various functions, including predator avoidance, circadian rhythms, phototaxis and migration. Here we confirm that planarians (like *Caenorhabditis elegans*, leeches and *Drosophila* larvae) are capable of detecting and responding to light using extraocular photoreception. We found that, when either eyeless or decapitated worms were exposed to near-ultraviolet (near-UV) light, intense wild-type photophobic behaviors were still observed. Our data also revealed that behavioral responses to green wavelengths were mediated by ocular mechanisms, whereas near-UV responses were driven by extraocular mechanisms. As part of a candidate screen to uncover the genetic basis of extraocular photoreception in the planarian species *Schmidtea mediterranea*, we identified a potential role for a homolog of the transient receptor potential channel A1 (*TRPA1*) in mediating behavioral responses to extraocular light cues. RNA interference (RNAi) to *Smed-TrpA* resulted in worms that lacked extraocular photophobic responses to near-UV light, a mechanism previously only identified in *Drosophila*. These data show that the planarian *TRPA1* homolog is required for planarian extraocular-light avoidance and may represent a potential ancestral function of this gene. TRPA1 is an evolutionarily conserved detector of temperature and chemical irritants, including reactive oxygen species that are byproducts of UV-light exposure. Our results suggest that planarians possess extraocular photoreception and display an unconventional TRPA1-mediated photophobic response to near-UV light.

## Abstract (precis)

**Summary:** Planarians use a non-traditional TRPA1-mediated mechanism for extraocular photoresponses to near-UV light, a response previously identified only in *Drosophila.*

## INTRODUCTION

The ability to detect and respond to light is a fundamental characteristic of living organisms. Ocular photoreception (or vision) is what is most commonly associated with light detection and image formation, an ability that requires central nervous system processing from cells found specifically in the eye organ. However, many animals also have the ability to detect light using light-sensitive structures outside of the eye. Such extraocular photoreception (also known as dermal phototransduction, dispersed photoreception or non-ocular photoreception) describes a type of ‘non-visual’ light detection that it is not involved in image formation.

Whereas the molecular basis of ocular phototransduction is studied extensively, the mechanisms involved in extraocular photoreception and transduction are not as well understood. This is despite the fact that the ability to detect light outside of the eye is widely distributed throughout the animal kingdom. Both vertebrate and invertebrate extraocular photoreception has been documented (Cronin and Johnsen, 2016; Lees, 1948; Porter, 2016; Steven, 1963). For example, mollusks and Cnidaria use extraocular photoreception for phototaxis and/or shadow-induced withdrawal (Lukowiak and Jacklet, 1972; Pankey et al., 2010; Ramirez et al., 2011; Taddei-Ferretti and Musio, 2000); leeches use extraocular photoreceptors for dorsal–ventral body orientation (Jellies, 2014); in amphibians, extraocular photoreceptors are required for detection of polarized light and magnetic orientation (Adler and Taylor, 1973; Phillips et al., 2001); whereas birds possess photoreceptors in the hypothalamus that regulate their circadian and reproductive cycles (Menaker, 1968).

The mechanisms involved in classical ocular phototransduction are well characterized and appear to be highly conserved throughout the Bilateria (Arendt, 2003). Phototransduction occurs when a photon of light activates a light-sensitive photopigment, which consists of a chromophore and an opsin (Wald, 1968). Opsins are G-protein-coupled receptors that are responsible for ocular light detection in all animals. Opsins are typically located within either rhabdomeric or ciliary photoreceptor cells, where they activate r-opsin or c-opsin signal transduction cascades, respectively (Arendt, 2003). C-opsins initiate a pathway that closes cyclic-nucleotide-gated (CNG) ion channels (Kaupp and Seifert, 2002), whereas r-opsins lead to the opening of transient receptor potential cation (TRPC) channels (Hardie, 2001). Both cascades result in signals that are interpreted by the brain to produce behavioral responses in the animal.

Although planarian eyes are simpler than vertebrate eyes, they still possess several phylogenetically conserved features. For example, eye development in many animals, including both planarians and vertebrates, relies on common genes, such as the homologs to *Sine oculis*, *Eyes absent* and *Otx* (Mannini et al., 2004; Martin-Duran et al., 2012; Pineda et al., 2000). Planarian eyes are located on the dorsal side of the body and consist of two cell types: pigment cells and photoreceptor cells. Pigment cells form a semi-lunar pattern within the optic cup and function to absorb photons of light, which creates shade for the photoreceptor cells, enabling directional information about incoming light (Nilsson, 2009). Photoreceptor cell bodies are found outside of the optic cup and project axons posteriorly to the brain, with some fibers forming a partial optic chiasma (Agata et al., 1998; Carpenter et al., 1974; Okamoto et al., 2005). Photoreceptor cell dendrites extend into the optic cup, making a rhabdomeric structure where opsin accumulates (Azuma and Shinozawa, 1998; Orii et al., 1998). Similar to rhabdomeric photoreceptors in other invertebrates, planarians express rhabdomeric transduction components, including two r-opsin orthologs, Gα-q, phospholipase C and two TRPC orthologs (Lapan and Reddien, 2012; Orii et al., 1998). Interestingly, transcriptome analysis has also shown that planarian eyes express genes that are typically associated with the phototransduction pathway found in ciliary photoreceptors, such as *CNG* (Lapan and Reddien, 2012). However, the roles of these genes in planarian vision are not currently known.

In contrast to ocular photoreception, the mechanisms used for extraocular photoreception have not been as extensively studied, and the few molecular pathways identified are more wide-ranging. Some animals appear to reuse the same ocular phototransduction receptors and pathways for extraocular photoreception. Cuttlefish and pond snails use c- and r-opsins, respectively, for extraocular photoreception (Mathger et al., 2010; Pankey et al., 2010), whereas Cnidarians use Gs-opsins (or ‘cnidops’), which, in *Hydra*, are believed to activate CNG channels (Plachetzki et al., 2010). Although poorly characterized, ‘RGR/Go-opsins’ are another group of opsins known to have extraocular function (Feuda et al., 2012; Porter, 2016; Raible et al., 2006).

In addition to these opsin-based mechanisms, a few other mechanisms unique to extraocular photoreception have been identified. Cryptochromes are ultraviolet (UV)- and blue-light-sensitive proteins that have been shown to regulate a variety of different light responses, including circadian rhythms in both plants and animals (Chaves et al., 2011; Haug et al., 2015) and magnetoreception (Bazalova et al., 2016; Gegear et al., 2008). There have also been pathways identified that center on gustatory-related receptor proteins. In *Caenorhabditis elegans*, two gustatory-related receptors, LITE-1 and GUR-3, have been found to elicit UV-light avoidance and together also inhibit feeding behavior (Bhatla and Horvitz, 2015; Edwards et al., 2008). Similarly to *C. elegans*, *Drosophila* larvae exhibit avoidance behavior to blue and UV light using the gustatory receptor gene *GR28b* (its closest homolog to *LITE-1*), which is found in the neurons that tile the body wall. This mechanism also involves the ion channel transient receptor potential A1 (TRPA1) (Xiang et al., 2010). The existence of such variable mechanisms for extraocular photoreception opens up questions about its evolutionary origins.

Furthermore, there is conflicting evidence for the existence of extraocular photoreception in certain species, as is the case for planaria. Planarians are free-living flatworms that make excellent models for investigating the basic features of eye biology and evolution due to their relatively simple yet phylogenetically conserved visual systems (Lapan and Reddien, 2012; Orii et al., 1998). Historical studies recorded the extraocular ability of planarians (along with most of the other aquatic animals that were surveyed) to respond to light (Steven, 1963). Early experiments that used surgical ablation to remove both eyes showed that eyeless planarians are negatively phototaxic and will change direction in response to white light (Parker and Burnett, 1900; Taliaferro, 1920). However, more recent studies that also specifically removed the eyes failed to observe any behavioral responses to white light (Arees, 1986; Azuma and Shinozawa, 1998). We hypothesize that planarians are in fact capable of extraocular photoreception, and that previous reports may have disagreed owing to the use of different sources of white light (which had different spectral compositions). White light is composed of many wavelengths, and our previous work has demonstrated that planarian behavioral responses vary by wavelength (Paskin et al., 2014). We set out to investigate whether planarians possess extraocular photoreception. Finding that planarians did respond to extraocular light cues, we then investigated whether this response was wavelength-specific and what possible genetic mechanisms might be involved.

## MATERIALS AND METHODS

### Animals and colony care

An asexual strain of *Schmidtea mediterranea* was used and maintained as previously described (Paskin et al., 2014), with worm water comprising 0.5 g l^−1^ Instant Ocean salts (Spectrum Brands, Blacksburg, VA, USA). Worms used were 7–9 mm in length and were starved for at least 1 week prior to experimentation.

### Light sources

Behavioral assays were conducted using commercially available red, green and near-UV laser pointers with nominal peak wavelengths of 650, 532 and 405 nm (±10 for all), respectively. A laser power meter (LaserBee A 2-Watt Laser Power Meter/Thermopile, J.BAUER Electronics, Canada) was used to determine the absorbed power for each laser: red=85 mW, green=29 mW and near-UV=54 mW. The power was then used to calculate the intensity (Watts/area of light) of each wavelength: red=0.68 W cm^−2^, green=0.23 W cm^−2^, near-UV=0.43 W cm^−2^. A piece of tape was placed on the end of the laser and punctured to create a pinhole that was smaller than the worm itself and produced a circle of light with a diameter of approximately 2.5 mm. The power of each laser with the pinhole was also examined but all were below the level of thermopile detection (<1 mW).

### Avoidance assay

Ocular responses were tested using an avoidance assay that we previously developed (Paskin et al., 2014). A 100 mm Petri dish filled with 20 ml of worm water was positioned over a white piece of paper and placed on the microscope stage. The white paper enables the laser light to be seen. The base bright-field light of the microscope was set to the lowest possible setting that allowed for video recording of worm position (∼275 lux). This was considered our ‘ambient’ light level, and all experiments were performed under this setting. Individual worms were transferred to the middle of the Petri dish and video recording was started when the worm began traveling in a straight line. The hand-held light source was introduced by a perpendicular approach that avoided the animal and directed a spot of light in front of the animal at a distance equal to one diameter of the circle of light (∼2.5 mm). The light was held stationary at that spot while the worm traveled. Recording was stopped after worms either passed through the light (no response) or responded (avoided the light). Worms were tested in order of decreasing wavelength (red, then green, then near-UV). For each wavelength, 30 worms were tested 4 times for a total of 120 trials per wavelength. Control ‘no light’ experiments were performed without the laser light cue being presented (30 worms were tested 3 times for a total of 90 control trials). The recording time for no light controls was 2.5 s (the average time required to elicit a behavioral response in a random sample of red, green and near-UV trials, plus 0.3 s).

Behavioral responses were determined as follows: no response (movement of the worm through any part of the light); moderate response (movement around the light at an angle less than 90 deg from the worm's original trajectory); and severe response (movement in the opposite direction of the light at an angle of 90 deg or greater). Because worms randomly explore new environments (i.e. do not always travel in a straight line), the amount of ‘responses’ (either moderate or severe) recorded in no light controls represents the level of background noise (random turning) in the assay. Significance was determined by calculating the percentage of worms that exhibited each of the 3 responses followed by a two-sample *t*-test between percentages using the Statistics Calculator software (StatPac, V. 4.0, StatPac Inc., Northfield, MN, USA) with *P*<0.0001 being considered as significant.

### Extraocular assay

Extraocular responses to light were tested using the microscope, Petri dish and laser pointer set-up as described for our avoidance assay. The same worms tested for ocular responses were tested for extraocular responses to allow for a comparison of ocular and extraocular responses in the same individual. As worms moved across the dish, the hand-held laser light was shone directly on the tail (midway between the tip of the tail and the pharynx), with the light introduced from behind the worm to avoid involvement of the eyes. The light's position on the tail was maintained by moving the laser light with the worm (so that the light remained on the tail) until after a response was observed or for 5 s if no response was observed. No light controls were recorded for 5 s. Behavioral extraocular responses were determined by the presence of tail thinning.

To assess tail thinning, we analyzed an image of the worm just before the light was positioned (‘Before’), as well as an image when the tail appeared thinnest (‘After’). When no thinning was apparent, the ‘After’ image used was at 3 s after the spot of light was positioned (the average time it took for peak thinning in animals with a response). The two pictures (‘Before’ and ‘After’) were then analyzed in Photoshop (Adobe Systems, San Jose, CA, USA) by measuring the width of the tail (in pixels) halfway between the most posterior part of the pharynx and the tip of the tail. Thinning responses were expressed as the percentage of the animal that had thinned: the width of the ‘After’ image was divided by the width of the ‘Before’ image, and this value was subtracted from 1. Significance between the average percentage thinned in no light control animals versus red, green or near-UV wavelengths was determined using a Student's *t*-test with *P*<0.0001 considered as being significant.

### Neutral density filters

Filters used were 25.4 mm diameter nickel–chromium-coated fused silica (7980) as previously described (Jellies, 2014). A holder was designed from a small PVC pipe to position the laser pointer above the filter such that all emitted light passed through the filter. Neutral density filters attenuating 75% of light (optical density=0.6), 95% of light (optical density=1.3) and 99% of light (optical density=2.0) were used. Significance between the average percentage thinned in animals exposed to full near-UV light versus near-UV attenuated light was determined using a Student's *t*-test with *P*<0.0001 being considered as significant.

### Worm fragment assay

Amputations were performed as previously described (Beane et al., 2013). 1/5 fragments (head, pre-pharynx, pharynx, post-pharynx and tail) were generated by cutting just posterior to the auricles, just anterior to the pharynx, just posterior to the pharynx, and midway between the pharynx and the tail. Fragments were transferred to non-treated tissue-culture welled plates, and worm water was changed immediately following surgery. After 1–2 h of recovery, fragments were tested for extraocular responses as described above with the following exceptions: only no light controls and near-UV laser light trials were performed (*n*=20 for each); instead of being hand-held, the near-UV laser pointer was positioned using a clamp stand approximately 2 inches above the worm, with the light positioned on the center of the fragment; and each fragment was recorded for 45 s or until it had moved out of the laser light, whichever occurred first.

To assess extraocular responses in fragments, an image when the light was first positioned (‘Before’) and an image when the worm first moved out of the field of light (‘After’) were analyzed. For no light controls, an image at 45 s was used for ‘After’. ‘Before’ and ‘After’ images from each fragment trial were overlaid in Photoshop, and the distance between the most posterior edge of the fragment in each image was measured (in pixels). Using this distance measurement and the time it took for the fragment to leave the light (or 45 s for control), the rate of movement was calculated. Significance was determined using a Student's *t*-test with *P*<0.001 being considered as significant.

### Eye ablation assay

Double eye ablations and sham ablations were performed as previously described (Deochand et al., 2016). After 24 h, behavioral responses to green and near-UV light were tested and analyzed using the avoidance assay described above. For each wavelength, *n*=50 for the sham-ablated group and *n*=30 for the double-eye-ablated group. Significance was determined using a two sample *t*-test between percentages using StatPac (V. 4.0) with *P*<0.05 being considered as significant.

### Cloning

Homologs to cyclic-nucleotide-gated channel A (*CNG-A*) and *LITE-1* (NP_509043.3) were used to search (tBlastn), the *S. mediterranea* Genome Database (Robb et al., 2008, 2015). To confirm identity, the resulting candidate sequences were used to search (tBlastx) NCBI (http://blast.ncbi.nlm.nih.gov/Blast.cgi). Previously identified planarian sequences to transient receptor potential cation channel, subfamily A (*Smed-TrpA*) (Wenemoser et al., 2012) and opsin homologs (Lapan and Reddien, 2012; Sanchez Alvarado and Newmark, 1999) were identified from the literature. An *S. mediterranea* cDNA library (from intact worms) was used to generate initial gene fragments by PCR with primers designed using Primer3plus (http://www.bioinformatics.nl/cgi-bin/primer3plus/primer3plus.cgi/). PCR fragments were ligated into pCRII-TOPO (Invitrogen, Carlsbad, CA, USA) and confirmed by sequencing. Protein domain analyses were performed using the NCBI Conserved Domains Database (http://www.ncbi.nlm.nih.gov/cdd) (Marchler-Bauer et al., 2015). Primer sequences used were: TrpA: Smed-TrpA forward 5′-CAACTCGACACCTTTGCACTA-3′; Smed-TrpA reverse 5′-CAACCTCCCAAATGAGTCTGT-3′. CNGA: Smed-CNGA3 forward 5′-GATTCAGAATGGATGCTT-3′; Smed-CNGA3 reverse 5′-TGTGCCAATTAAAACTCC-3′; Smed-CNGA3-Like forward 5′-AACATTCTCGTGAATCGGAAC-3′; Smed-CNGA3-Like reverse 5′-TAACTCCCAAATTCGTTCTGG-3′. Opsin: Smed-opsin-Homolog-1 forward 5′-TCTTTTGGTTTTGGTGGACAG-3′; Smed-opsin-Homolog-1 reverse 5′-TCCATCAACACAATGGCACTA-3′; Smed-opsin-Homolog-2 forward 5′- GGTTTCATCGGTGGTCTTTT-3′; Smed-opsin-Homolog-2 reverse 5′-ACCCGTTTTCATGGAAGTTG-3′.

### RNA interference (RNAi)

RNAi was performed as previously described (Rouhana et al., 2013). In summary: double-stranded RNA (dsRNA) was generated by using the above pCRII-TOPO constructs to make linearized templates via PCR (using T7 and SP6 primer sequences). This PCR template was used for *in vitro* dsRNA synthesis with T7 and SP6 RNA polymerases (Promega P2075, P1085, N2511, P1221, M6101; Promega, Madison, WI, USA). An RNAi mixture of 100 ng/μl in liver puree (Creekstone Farms, Arkansas City, KS, USA) plus 1% red food coloring was made. Worms were fed RNAi in Petri dishes (5 μl per worm) 3 times over 8 days before being used on day 14 (from first feeding) to test behavioral responses as described above (avoidance and extraocular assays). Significance was determined for avoidance trials using a two-sample *t*-test between percentages, with *P*<0.05 being considered as significant. For the extraocular assay, a one-way ANOVA with Tukey's multiple comparisons test, with *P*<0.0001, was used for significance.

### *In situ* hybridization

Whole-mount *in situ* hybridization was performed as described in Pearson et al. (2009), with modifications as described in Deochand et al. (2016) except that samples were incubated in formamide-bleaching solution for 4 h as described in King and Newmark (2013). The *Smed-TrpA* probe was used at 4 ng μl^−1^. Anti-digoxigenin-AP (Roche, Basel, Switzerland) was used at 1:3000.

### Image collection

All images were collected using a Zeiss V20 fluorescent stereomicroscope with AxioCam MRc or MRm camera and Zen Lite software (Zeiss, Oberkochen, Germany).

## RESULTS

### Planarians possess both ocular and extraocular responses to light

Planarian behavioral responses to light are complex. Dorsal eye spots (ocelli) regulate a strong photophobic avoidance across a wide spectrum of light wavelengths (Brown et al., 1968; Paskin et al., 2014). Additionally, studies have suggested that planarians possess the ability to respond to light via extraocular mechanisms and will display avoidance behaviors following surgical removal of the eyes (Parker and Burnett, 1900; Taliaferro, 1920). Our previous research has shown that different wavelengths elicit different behavioral responses in planarians (Paskin et al., 2014). However, these studies did not separate out any contribution that may have been made by extraocular photoreception to the behaviors observed. Therefore, we modified our previously described light-avoidance assay (Paskin et al., 2014) in order to investigate extraocular responses to different wavelengths of light.

**Fig. 1.**
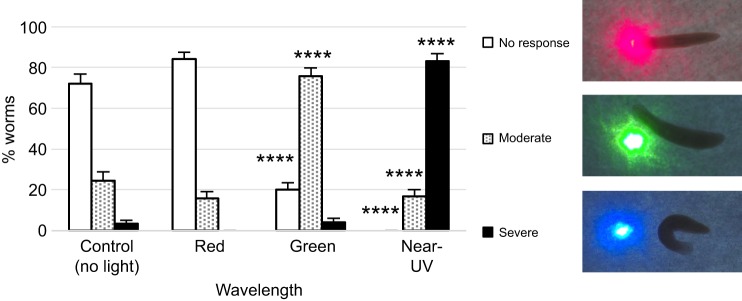
**Planarians possess complex ocular photoresponses.** The planarian species *Schmidtea mediterranea* was used to examine ocular behavioral responses to red, green and near-UV wavelengths using our avoidance assay. When approaching a spot of light, three distinct behaviors are observed: worms move through the light (no response), worms avoid the light by moving around it (moderate response) or worms make a 90–180 deg turn away from the light (severe response). Note that worms fail to respond to red light, display moderate responses to green light and have severe responses to near-UV light. *N*=120 for red, green and near-UV, *N*=90 for control. Statistics: two sample *t*-test between percentages; ****P<0.0001 (as compared with no light controls); error bars=s.d.

We set out to test both ocular and extraocular behavioral responses in the same individuals. To measure ocular responses to light, we performed our avoidance assay where a point of red, green or near-UV laser light is placed directly in front of a worms' path (Fig. 1) (Paskin et al., 2014). Planarians display three distinct behaviors: (1) no response, with continued movement directly through the light; (2) a moderate response, with a directional change to avoid the light; and (3) a severe response, with an abrupt turn of ≥90 deg away from the light. Negative controls, with no laser light, were also performed (Fig. 1). Consistent with our previous findings, the majority of worms (>80%) had no response to red light (which was not significantly different from controls), whereas 75.83% displayed a moderate response to green light and >80% displayed a severe response to near-UV light (Fig. 1). The near-UV severe response is so penetrant that no animals traveled through the near-UV light (100%). These results demonstrate that this population of planarians responded as predicted, with wild-type reactions to each different wavelength of light (and the shorter near-UV wavelengths causing the strongest photophobic reactions).

**Fig. 2.**
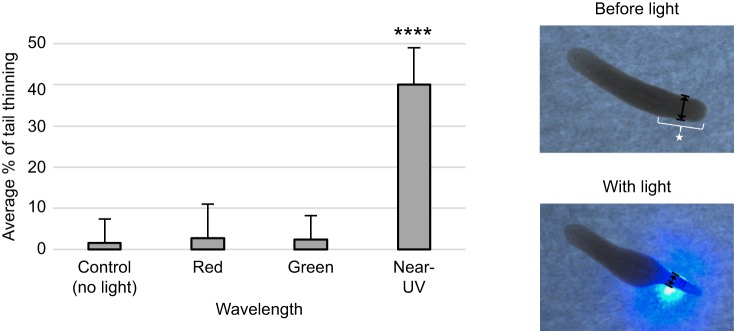
**Planarians possess extraocular photoreception.** Graph of extraocular responses to light using our extraocular assay. A spot of light was placed on the worm's tail, and the presence of the photophobic ‘tail thinning’ response was assessed. Thinning was determined by measuring the width of the tail (midway between the posterior edge of the pharynx and tip of tail) both before and after exposure to red, green and near-UV laser light. Photos: starred bracket designates the tail; double-headed black arrows designate the width measurement. Note that significant tail thinning was observed only with near-UV wavelengths. *N*=40 for control, red, and green; *N*=120 for near UV. Statistics: two-tailed independent *t-*test; ****P<0.0001 (as compared with no light controls); error bars=s.d.

Having established this baseline, we next used these same animals to examine extraocular behavioral responses. For our extraocular assay, either red, green or near-UV laser light was placed directly on the animals' tail (Fig. 2). The same diameter of light used in our avoidance assay was positioned on the most posterior part of the worm (the tail) without ever illuminating the head or eyes. Using this method, we observed responses to some extraocular light sources of a ‘thinning’ of the tail (Fig. 2), presumably to reduce the surface area exposed to the light source, followed by swift movement (pulling of the tail) out of the spot of light. This response was analyzed by measuring the width of the tail halfway between the most posterior part of the pharynx and the tip of the tail (star in Fig. 2) and was expressed as the percentage of tail thinned. We observed no behavioral responses to either red or green wavelengths (Movie 1), with responses not being significantly different from the no light controls (*P*≥0.45, Fig. 2). However, exposure to near-UV light resulted in a marked thinning of the tail (Movie 1), with an average decrease in tail width of 40.15% (*P*<0.0001, Fig. 2). These results demonstrate that planarians are in fact capable of extraocular photoreception and furthermore that their extraocular light detection is specific to near-UV wavelengths.

**Fig. 3.**
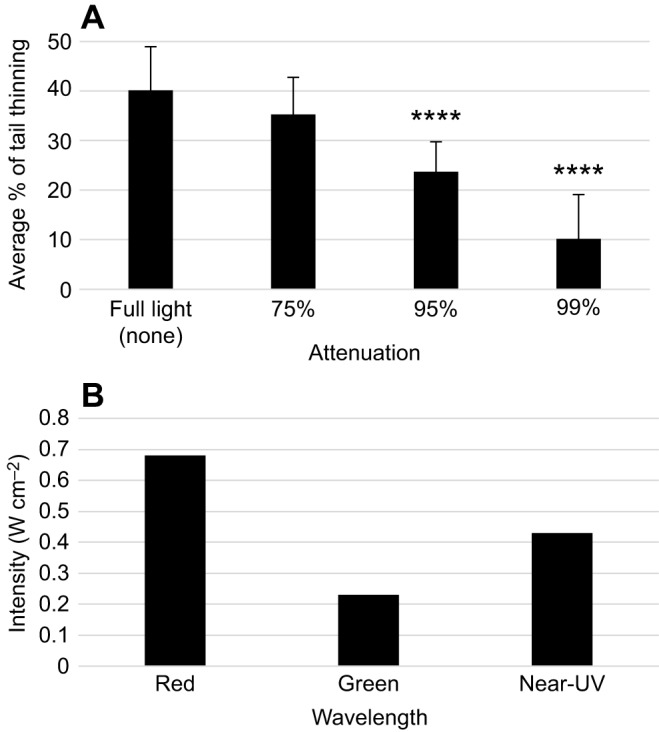
**Extraocular behavioral responses result from detection of near-UV light stimulus.** (A) Graph showing extraocular behavioral responses with increasingly attenuated light, as measured by amount of tail thinning when exposed to near-UV laser light (*n*≥40). Worms were exposed to full light, and 75%, 95% and 99% attenuated light (or optical densities of 0, 0.6, 1.3 and 2.0, respectively). The trend shows that extraocular behaviors decrease with diminished light stimulus. Statistics: two-tailed independent *t-*test; ****P<0.0001 (as compared with full light responses); error bars=s.d. (B) Graph showing red, green and near-UV laser light intensities (W cm^−2^). The light intensities do not correlate with planarian tail thinning because the most intense light, red, elicits no response.

We next wanted to determine whether any confounding variables might be contributing to the behavioral responses we observed. First, we repeated the extraocular assay using near-UV light in combination with neutral density filters to determine whether or not there was a linear correlation between the light source and the behaviors observed (Fig. 3A). Because neutral density filters attenuate light, which is our relevant stimulus, we would expect tail thinning to decrease in correlation with an increase in light attenuation. In the first trial, the near-UV laser light was attenuated by 75%, meaning that only 25% of the light reached the animal. For the second trial 95% of the light was attenuated, whereas, in the last trial, the near-UV light was attenuated by 99%. Our results revealed a steady decrease in behavioral responses to near-UV light (tail thinning) with increased light attenuation, such that, with both 95% and 99% attenuation, tail thinning was significantly less than full-power near-UV light controls (Fig. 3A). Furthermore, there was a significant decrease in responses between each neutral density filter trial (*P*≤0.01). These data confirm that extraocular responses to near-UV light diminish in a predictable fashion as light attenuation increases. Second, we used a laser power meter and confirmed that the laser light emitted from the pinhole for each wavelength produced very little power (and therefore heat), with levels below the threshold of the thermopile (<1 mW). These data suggest that heat was not a factor involved in the behavioral responses observed. Finally, we calculated the intensities of the full power of each laser light (no pinhole) to determine whether or not light intensity differences between the wavelengths were a confounding variable. Our data showed no correlation between the light intensity of each wavelength and the corresponding behavioral response (or lack thereof). In fact, the red laser pointer actually produced the most power per unit area (Fig. 3B), even though worms had no response to red light (Figs 1 and 2). Together, these data suggest that the behavioral responses observed (tail thinning) were attributable to near-UV-light detection.

### Extraocular light responses occur across the entire body

Our extraocular assay showed that the post-pharyngeal tissues of the tail possess extraocular photoreception. However, the nature of the assay (using whole worms) means that we could not rule out the possibility that animals were still receiving a small amount of ocular input, which could be contributing to the observed response. Furthermore, our previous assay did not allow us to evaluate whether the extraocular response to near UV occurs along the entire anterior–posterior axis of the worm (as opposed to being confined to just the tail region). Planarians have the ability to survive and regenerate when cut into multiple fragments, including the movement of new fragments lacking any brain tissues in response to stimuli (Beane et al., 2011). We used this unique planarian characteristic to perform a worm fragment assay to confirm that extraocular responses do not require the eyes, as well as to examine whether extraocular responses also occur in other regions of the body.

**Fig. 4.**
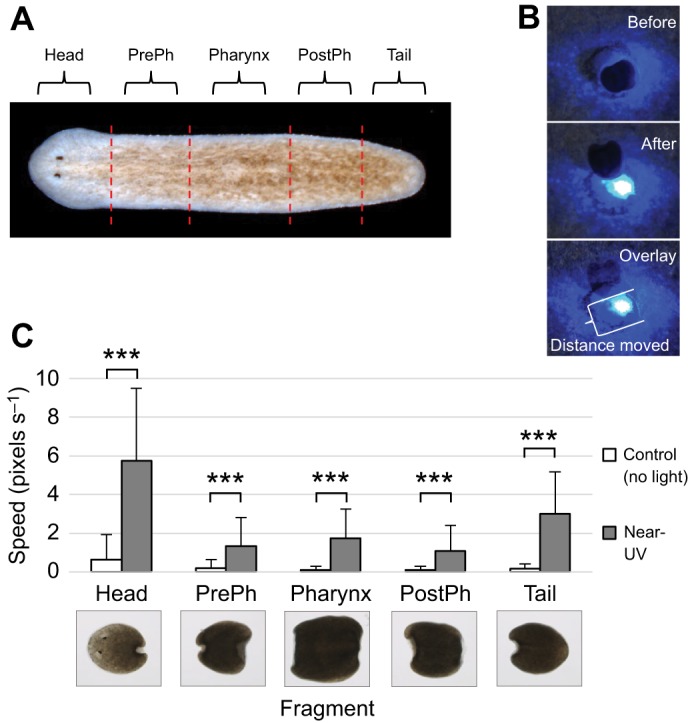
**Extraocular photoreception occurs along the entire body.** Worm fragment assay, in which worms were cut into 5 fragments along the anterior–posterior axis and individual fragments were tested at 1–2 h post-amputation for extraocular photophobic behavioral responses to near-UV light. (A) Diagram of the amputation scheme and resulting fragments. (B) Photophobia was assessed by analyzing the speed of fragments moving out of the cone of light [distance moved by the most posterior edge of the fragment (bracket) divided by time]. (C) Graph of near-UV-light avoidance in worm fragments, showing that near-UV light causes a significant increase in speed for all fragments (*n*=20). PrePh, pre-pharyngeal; PostPh, post-pharyngeal. Statistics: two-tailed independent *t-*test; ***P<0.001 (as compared with no light controls); error bars=s.d.

For our worm fragment assay, each worm was cut into 5 sections: head fragments, pre-pharynx fragments, pharynx fragments, post-pharynx fragments and tail fragments (Fig. 4A). Because new worm fragments do not move as much as whole worms (and typically not without a stimulus), we modified our extraocular assay and analyses to accommodate fragments. Behavioral responses for each fragment were recorded for 1 min with no laser light (controls) and again with near-UV laser light with the spot of light placed directly in the center of each fragment (Fig. 4B). From these data, we calculated the speed at which each fragment moved out of the near-UV light, by using the time and distance the fragment had moved (Fig. 4B). Our results demonstrate that, whereas control fragments (with no light stimulus) moved very little (as expected), exposure to near-UV light caused a significant increase in speed for all fragments tested (*P*≤0.001, Fig. 4C). These data show that extraocular responses to near-UV occur across the entire body of the planarian. Additionally, our results suggest that detecting and responding to near-UV light does not require either ocular input or the brain.

### Extraocular behavioral responses require TRPA1

Together, our data from the extraocular assay and worm fragment assay demonstrate that planarians are capable of extraocular detection of light. Furthermore, our results show that these responses are specific to near-UV wavelengths and occur along the entire anterior–posterior axis of the animal. However, the genetic mechanism(s) for extraocular photoreception in planarians are unknown. We took a candidate gene approach to uncover potential mechanisms by searching the *S. mediterranea* genome (Robb et al., 2008, 2015) and the literature for planarian homologs to genes that regulate extraocular photoreception in other animals: those encoding CNG channels, opsin, Lite-1 and TRPA1 (Bhatla and Horvitz, 2015; Edwards et al., 2008; Mathger et al., 2010; Pankey et al., 2010; Plachetzki et al., 2010; Xiang et al., 2010). We found no potential homologs for the *C. elegans Lite-1* gene; however, *S. mediterranea* homologs for the other extraocular photoreception genes were identified (Table 1). Therefore, using RNAi, we examined the role of the two planarian opsin orthologs (Lapan and Reddien, 2012; Sanchez Alvarado and Newmark, 1999), two *CNG-A* homologs and the *TRPA1* homolog (*Smed-TrpA*) (Wenemoser et al., 2012) in mediating planarian extraocular responses.

**Table 1.**
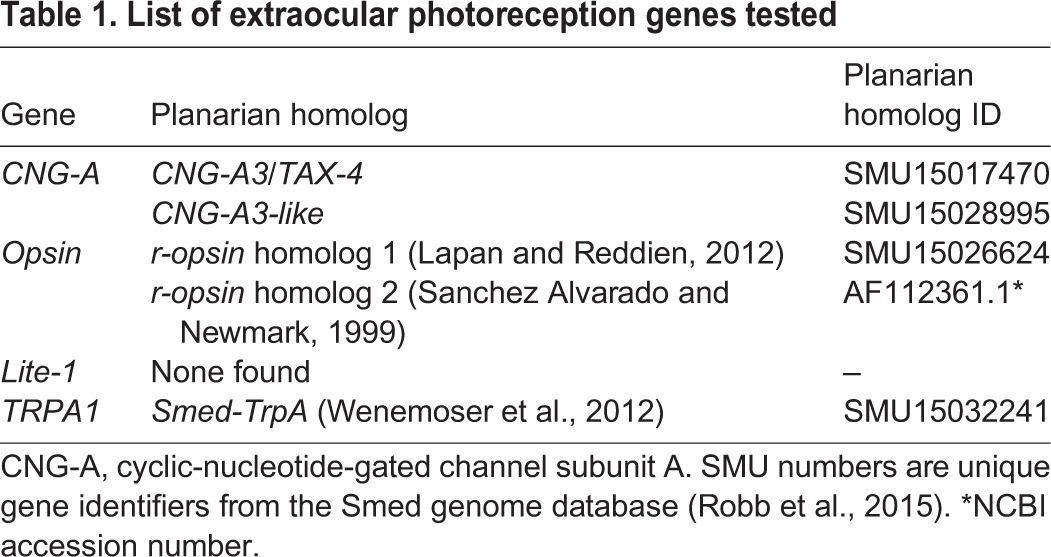
**List of extraocular photoreception genes tested**

**Fig. 5.**
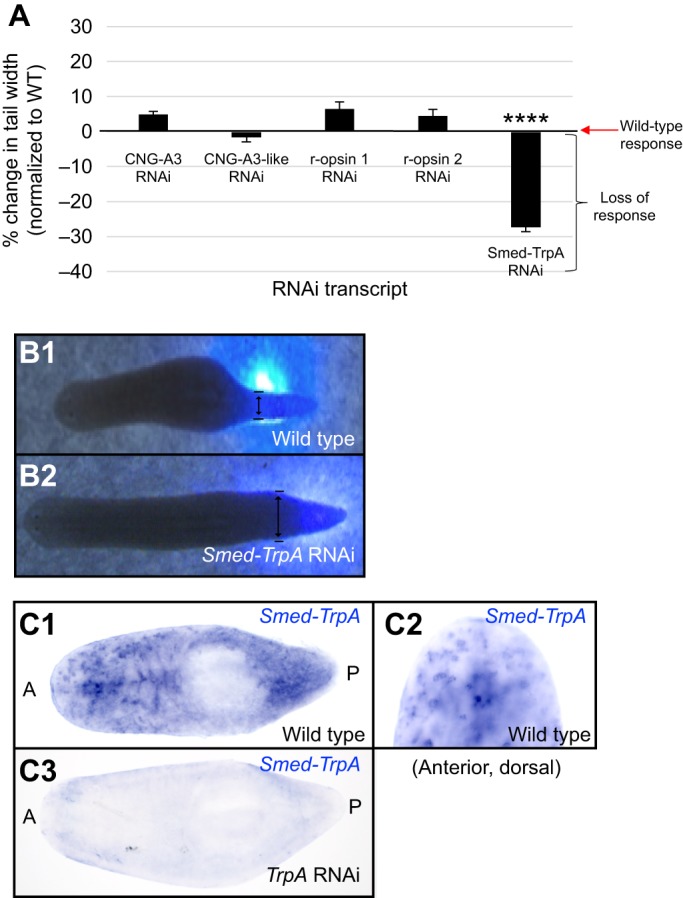
***Smed-TrpA* is required for extraocular behavioral responses to near-UV light.** (A) Graph of extraocular behavioral responses to near-UV light following RNA interference (RNAi). Genes known to regulate extraocular photoreception in other animals (homologs to the genes encoding CNG channels, opsins and TRPA1) were knocked down following double-stranded RNA (dsRNA) feeding then tested for tail-thinning response. Graph is normalized to wild-type response. Tail thinning was significantly decreased only in *Smed-TrpA(RNAi)* animals (*N*≥21). Statistics: one-way ANOVA with Tukey's multiple comparisons test; ****P<0.0001 (as compared with wild-type responses); error bars=s.e.m. (B) Images showing wild-type thinning response (B1) and lack of response in *Smed-TrpA(RNAi)* animals (B2). Double-headed black arrows designate the width measurement. (C) *In situ* hybridization for *Smed-TrpA*. Wild-type worms express *Smed-TrpA* along the entire length of the planarian body (C1), particularly in dorsal tissues (C2), whereas expression is lost following *Smed-TrpA(RNAi)* feeding (C3). *N*≥13. A, anterior; P, posterior.

Treating whole worms with dsRNA made to the 5 identified homologs from Table 1, we found that only *Smed-TrpA(RNAi)* influenced extraocular behavioral responses to near-UV wavelengths (Fig. 5A). TRPA1 is an ion channel that has been shown to be required for extraocular photoreception of UV light in *Drosophila* larvae (Xiang et al., 2010). Protein domain analysis shows *Drosophila* TRPA1 and planarian Smed-TrpA to be highly conserved (data not shown). In our extraocular assay using near-UV laser light, the amount of tail thinning in worms in which the homologs to *CNG-A* and *r-opsin* were knocked down by RNAi were not significantly different from wild type (Fig. 5A). However, *Smed-TrpA(RNAi)* animals showed a significant decrease in tail thinning (Fig. 5A,B2, Movie 1), as compared with wild-type worms (*P*<0.0001, Fig. 5A,B1). These data suggest that, similar to *Drosophila* larvae, TRPA1 is required for planarian extraocular behavioral responses to near-UV wavelengths. *In situ* hybridization of *Smed-TrpA* transcripts revealed that planarian TRPA1 is expressed throughout the entire anterior–posterior axis (Fig. 5C1). These data are consistent with our worm fragment assay findings that planarians possess extraocular photoreception along their entire body. Furthermore, punctate *Smed-TrpA* expression was observed in dorsal tissues (Fig. 5C2), reminiscent of the dorsal (sub)epidermal expression patterns of planarian body pigment synthesis genes, such as *KMO-1*, *ALAS*, *ALAD-1* and *PBGD-1* (Stubenhaus et al., 2016). These data suggest that *Smed-TrpA* is in the right place to mediate planarian extraocular behavioral responses.

**Fig. 6.**
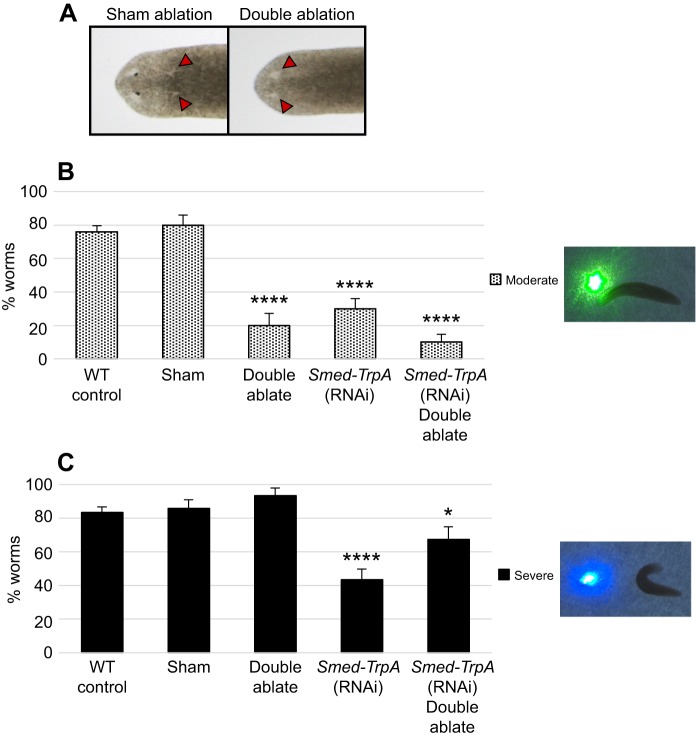
**Both ocular and extraocular behavioral responses involve *Smed-TrpA.*** (A) Representative examples of eye ablation assay. Sham controls had tissue removed posterior to the eyes, whereas eyes were surgically removed in experimental groups (double-eye ablation). Both groups were tested 24 h post-surgery. Red arrows: resected tissue. (B,C) Graphs showing avoidance responses to green (B) and near-UV (C) laser light across different treatment groups (*N*≥30). Double-eye-ablated worms showed a significant decrease in responses to green light, whereas near-UV-light avoidance remained similar to controls, suggesting that eyes are needed for photoreception of green (but not near-UV) wavelengths. *Smed-TrpA(RNAi)* significantly reduced responses to both green and near-UV light, suggesting a role for *Smed-TrpA* in both ocular and extraocular behavioral responses. Statistics: two sample *t*-test between percentages. ****P<0.0001, *P<0.05 (as compared with wild-type responses); error bars=s.d.

To more closely assess the role of *Smed-TrpA* in mediating extraocular versus ocular photoreception, we compared ‘blind’ (double eye ablated) animals to *Smed-TrpA(RNAi)* animals using our avoidance assay (Fig. 6). For these experiments, we used an eye ablation technique we previously developed that removes the eyes without disturbing the underlying brain tissues (Deochand et al., 2016). We found that sham surgery controls (where two pieces of anterior tissue outside the eye field were excised) displayed similar responses to uninjured wild-type worms for both green (*P*=0.98, Fig. 6B) and near-UV (*P*=0.67, Fig. 6C) wavelengths (see Movies 2 and 3 for wild-type responses). As expected, double-eye-ablated animals had significantly reduced responses to green light (*P*<0.05, Fig. 6B, Movie 2). Double ablation of the eyes had no effect on the worm's ability to respond to near-UV light as compared with wild-type animals (*P*=0.32, Fig. 6C, Movie 3). These data are consistent with our findings that planarians possess extraocular photoreception of near-UV wavelengths.

We found that *Smed-TrpA(RNAi)* animals had significantly reduced responses to both green and near-UV light (*P*<0.05; Fig. 6B,C; Movies 2 and 3). Our finding that, unlike wild type, *Smed-TrpA(RNAi)* animals largely failed to respond to green wavelengths in our avoidance assay (typically used to measure ocular responses) was unexpected, given that our data showed that planarians have no extraocular responses to green light (Fig. 2). Furthermore, responses to green light after double eye ablation of *Smed-TrpA(RNAi)* animals were not significantly different from responses after double eye ablation alone (*P*=0.24, Fig. 6B). These data suggest the possibility that behavioral responses to ocular photoreception may be mediated in part by *Smed-TrpA*. Interestingly, both CNG-A3 and CNG-A3-like were required for ocular behavioral responses (*n*=30, *P*≤0.05), whereas lack of r-opsin 1 or r-opsin 2 individually did not affect ocular behavioral responses (*n*=21, *P*≥0.05). These data suggest that the r-opsins have redundant functionalities, whereas the CNG channels have non-redundant functions during ocular responses. Together, our ablation assay data suggest that planarian responses to green light are largely driven by ocular photoreception, whereas behavioral responses to near-UV light are largely driven by extraocular photoreception. In summary, our data demonstrate that *Smed-TrpA* is required for behavioral responses to light, and specifically extraocular responses to near-UV light, in planarians.

## DISCUSSION

Our results support the hypothesis that planarians are in fact capable of extraocular photoreception and that light detection occurs along the entire body. Furthermore, similar to *Drosophila* larvae and *C. elegans*, extraocular photoreception in planarians is specific to near-UV wavelengths. We found that extraocular exposure to either red or green wavelengths did not elicit photophobic responses, unlike the significant tail thinning that was observed when planarians were exposed to near-UV light. In addition to our behavioral studies, we also discovered that *Smed-TrpA* is involved in planarian extraocular avoidance behavior to near-UV light. Like in *Drosophila* larvae, our results suggest that a TRPA1 ion channel homolog is required for wild-type tail thinning responses in planarians, because the normal photophobic responses to near-UV light are significantly decreased when *Smed-TrpA* is knocked down.

TRPA1 is a nonselective cation channel that is permeable to Ca^2+^, K^+^ and Na^+^ ions, and is a member of the large TRP family of ion channels. TRPA1 has been found in a variety of vertebrates and invertebrates, including humans, mice, rats, dogs, chickens, zebrafish, snakes, frogs, fruit flies, planarians and *C. elegans* (Inoue et al., 2014; Laursen et al., 2015; Nilius et al., 2012)*.* TRPA1 is unique in that it functions mainly to detect signals that cause pain and inflammation, such as from noxious chemicals and both mechanical and thermal stimuli (Bautista et al., 2013; Hill and Schaefer, 2009; Kwan et al., 2006; Zygmunt and Högestätt, 2014). It has also been determined that TRPA1 is activated in response to reactive electrophiles (which are tissue-damaging agents with aversive effects in both invertebrates and vertebrates), an activity that has been highly conserved for ∼500 million years (Kang et al., 2010). Electrophiles that activate TRPA1 are incredibly diverse and range from chemicals found in mustard and cinnamon to formaldehyde and acrolein, the latter of which is found in tear gas and vehicle exhaust emissions. In addition to external irritants, TRPA1 is also sensitive to endogenous agents such as reactive oxygen species (ROS) that are released by cells in response to tissue damage and inflammation (Bautista et al., 2013; Bessac and Jordt, 2008; Viana, 2016). Some of the ROS known to be TRPA1 activators include hypochlorite, hydrogen peroxide (H_2_O_2_) and ozone (O_3_) (Takahashi and Mori, 2011).

We found that, whereas planarians possess photophobic ocular responses to green light, they display no extraocular responses to green light. Although the majority of double-eye-ablated ‘blind’ animals had no response to green light, a small percentage were still able to respond. Although this avoidance could have been the result of residual eye tissue after surgery, planarians may also possess different types of extraocular photoreceptors in the head and tail. Interestingly, the majority of *Smed-TrpA(RNAi)* animals also had no response to green light (Fig. 6). These data suggest that TRPA1 is required for ocular behavioral responses to green light. This would appear to be the first recorded instance of TRPA1 involvement in ocular (visual) behavioral responses, although it does not rule out the possibility of off-target or compensatory effects. In addition, our data reveal that *Smed-TrpA* is required for extraocular responses specifically to near-UV wavelengths. Light-initiated behavioral responses (whether ocular or extraocular) involve photon capturing and phototransduction of light information to the nervous system (signal input), as well as translation of that input into specific behaviors (signal output). The data presented here do not distinguish between a role for *Smed-TrpA* in actual phototransduction as opposed to a role in the signal output controlling behavior.

Although our data does not exclude the possibility that *Smed-TrpA* is involved in converting photons into electrical signals (traditional phototransduction), alternative mechanisms have been proposed in both *Drosophila* larvae and human melanocytes. It has long been known that UV light exposure generates cellular ROS, including H_2_O_2_; additionally, there is now evidence linking UV-light-induced H_2_O_2_ production and activation of TRPA1 channels (Hill and Schaefer, 2009; McCormick et al., 1976). *Drosophila* larvae are capable of extraocular photoreception of UV light using cells found along their body wall (Xiang et al., 2010). A subsequent study identified two *Drosophila* TRPA1 isoforms that are directly activated by UV-produced H_2_O_2_ (Guntur et al., 2015). Similarly, it has been shown in humans that epidermal melanocytes detect UV light (resulting in melanin synthesis), where phototransduction appears to involve a G-protein-coupled receptor cascade that activates downstream TRPA1 channels (Bellono et al., 2014). These data implicate TRPA1 in mediating light-induced responses downstream of light detection.

The results of our neutral density filter experiments show that there is an inverse relationship between light attenuation and extraocular behavioral responses to near-UV light. We observed a steady decrease in tail thinning as light attenuation increased. We ruled out the possibility of contributions by either heat or differences in light intensity, suggesting that it is the light stimulus itself causing the behavioral responses. However, our data do not eliminate the possibility that the animals are responding to pain, or nociception. Given that UV light can cause detrimental biological effects and TRPA1 is known to respond to reactive electrophiles, including UV-induced H_2_O_2_, it is possible that planarian extraocular behavioral responses to near-UV light could be due to nociception.

Sensitivity to UV light is common in the animal kingdom, with its function ranging from mate selection in birds to feeding behavior in fish (Cronin and Bok, 2016; Hunt et al., 2001a,b). It has also been suggested that, in zooplankton (mainly crustaceans and some mollusks), avoidance of UV radiation is the driving force of diel vertical migrations (Gehring and Rosbash, 2003). A range of other invertebrates also display negative phototaxis to UV light, including *Daphnia*, *C. elegans*, *Drosophila* larvae and planarians (Edwards et al., 2008; Paskin et al., 2014; Storz and Paul, 1998; Xiang et al., 2010). It is well known that UV light causes significant damage to nucleic acids and proteins (Sinha and Häder, 2002). In planarians, extended exposure to UV radiation also causes damage to their protective mucosal layer and leads to visible wounds (Kalafatić et al., 2006). Therefore, in animals like planarians that have few natural defenses, avoidance of UV light might offer significant adaptive advantages.

In the current study, our results clearly demonstrate that planarians are indeed capable of extraocular photoreception. Conversely, a few studies have reported that they failed to observe extraocular behavioral responses in planarians (Arees, 1986; Azuma and Shinozawa, 1998), despite several accounts of planarian extraocular photoreception in the historical literature (Parker and Burnett, 1900; Steven, 1963; Taliaferro, 1920). The discrepancy between our results (demonstrating extraocular responses) and those that reported a lack of extraocular responses could be due to several factors. First, these other studies used different planarian species, specifically in the genus *Dugesia*, whereas our study examined *S. mediterranea.* Therefore, the observed differences could be merely species-related. However, because *Schmidtea* and *Dugesia* are closely related, a more likely explanation would be differences in the light source(s) used. Our results show that extraocular photoreception is specific to near-UV wavelengths and that planarians do not respond to longer wavelengths without eyes. These previous studies examining extraocular responses have used white light only, which is a combination of many different wavelengths, whose composition varies widely between light sources. Therefore, it is impossible to know the exact composition of wavelengths used from each study. Thus, the most likely explanation is that the white light source used in those early, historical experiments may have contained a greater percentage of UV wavelengths than the more recent studies.

Our data suggest that, similarly to *Drosophila*, extraocular near-UV light avoidance in planarians is mediated by TRPA1. This opsin-independent mechanism for extraocular photoresponses is intriguing because it suggests a separate evolutionary origin from opsin-based phototransduction. Additionally, the fact that several other extraocular mechanisms seem to be sensitive to UV light, including cryptochromes and the *C. elegans* gustatory-related receptors (Bhatla and Horvitz, 2015; Chaves et al., 2011; Edwards et al., 2008; Haug et al., 2015), might reflect the evolution of early life in aquatic environments where short wavelengths penetrate water more substantially than long wavelengths (Gehring and Rosbash, 2003). However, a true understanding of the evolution of extraocular photoreception will require investigation into the mechanisms in many other species, both among different planarian species as well as in other invertebrates and vertebrates.
